# Mitochondria Related Pathway Is Essential for Polysaccharides Purified from *Sparassis crispa* Mediated Neuro-Protection against Glutamate-Induced Toxicity in Differentiated PC12 Cells

**DOI:** 10.3390/ijms17020133

**Published:** 2016-01-26

**Authors:** Shuang Hu, Di Wang, Junrong Zhang, Mengyan Du, Yingkun Cheng, Yan Liu, Ning Zhang, Di Wang, Yi Wu

**Affiliations:** 1 School of Life Sciences, Jilin University, Changchun 130012, China; summerhu1990@outlook.com (S.H.); xiaowangdi@outlook.com (D.W.); archer707@163.com (J.Z.); jludumy@126.com (M.D.); chengyk@jlu.edu.cn (Y.C.); liuyaorui@126.com (Y.L.); zhangning1313@mails.jlu.edu.cn (N.Z.); 2 School of Pharmaceutical Science, Jilin University, Changchun 130021, China

**Keywords:** *Sparassis crispa*, purified polysaccharides, neuro-protection, glutamate, mitochondria

## Abstract

The present study aims to explore the neuro-protective effects of purified *Sparassis crispa* polysaccharides against l-glutamic acid (l-Glu)-induced differentiated PC12 (DPC12) cell damages and its underlying mechanisms. The *Sparassis crispa* water extract was purified by a DEAE-52 cellulose anion exchange column and a Sepharose G-100 column. A fraction with a molecular weight of 75 kDa and a diameter of 88.9 nm, entitled SCWEA, was obtained. SCWEA was identified with a triple helix with (1→3)-linked Rha in the backbone, and (1→2) linkages and (1→6) linkages in the side bone. Our results indicated that the pre-treatment of DPC12 cells with SCWEA prior to l-Glu exposure effectively reversed the reduction on cell viability (by 3-(4,5-cimethylthiazol-2-yl)-2,5-diphenyl tetrazolium bromide (MTT) assay) and reduced l-Glu-induced apoptosis (by Hoechst staining). SCWEA decreased the accumulation of intracellular reactive oxygen species, blocked Ca^2+^ influx and prevented depolarization of the mitochondrial membrane potential in DPC12 cells. Furthermore, SCWEA normalized expression of anti-apoptotic proteins in l-Glu-explored DPC12 cells. These results suggested that SCWEA protects against l-Glu-induced neuronal apoptosis in DPC12 cells and may be a promising candidate for treatment against neurodegenerative disease.

## 1. Introduction

Neurodegenerative disorders, which include Alzheimer’s disease (AD), Parkinson’s disease (PD) and amyotrophic lateral sclerosis (ALS), have received great attention due to their devastating nature and limited treatment options. During the process of neurological disorders, neurons manifest common pathological features, such as mitochondrial dysfunction and protein aggregation [1]. Although the underlying mechanisms of neurodegenerative diseases remain largely unknown, the idea that apoptosis of neurons plays a central role in the development of neurodegeneration has emerged as an attractive theory [2].

As one of the pathological factors in cerebral ischemia disease, glutamate is able to induce neuronal cell apoptosis via opening mitochondrial permeability transition pores (mPTP) and reducing mitochondrial membrane potential (MMP), which is initiated by the interaction between pro- and anti-apoptotic B-cell lymphoma 2 (Bcl-2) family members [3]. As a primary downstream mediator of phosphatidylinositol-3 kinase (PI3K), Protein Kinase B (AKT) preserves mitochondrial integrity by regulating the Bcl-2 family [4]. Reactive oxygen species (ROS) are responsible for later mitochondrial events, which lead to full activation of the caspase cascade [5]. In addition, the Ca^2+^ influx is involved in glutamate-induced cell apoptosis by mediating the opening of mPTP [6].

Extracts of herbs or fungi have been applied for centuries to elicit biological responses in patients. The acquisition of bioactive single components from natural extracts has become one of the main aims of drug research and development in biopharmaceutics. It has been found that polysaccharides extracted from different species of mushrooms and some other fungi were neuro-protective [7]. According to the study, polysaccharides isolated from the flowers of *Nerium indicum* exerted protective effects in cortical neurons induced by β-amyloid (Aβ) peptides [8]. Another polysaccharide fraction named J6, isolated from the flowers of *Nerium indicum*, showed a neuro-protective effect, which was associated with the inactivation of the c-Jun N-terminal kinase (JNK) signaling pathway [9]. Molecular weight, degree of branching, chemical composition and glycosidic linkages of polysaccharides are believed to be associated with their bioactivities [10].

*Sparassis crispa*, an edible mushroom with various medicinal properties, has been studied for years. As one of the main bioactive components, *Sparassis crispa* polysaccharides are reported to possess anti-tumor effects in mice with strong vascular dilation and hemorrhage reactions [11]. Oral administration of purified polysaccharides obtained from *Sparassis crispa* enhances the Th1 response on tumor-bearing mice [12]. On the other hand, *Sparassis crispa*–purified polysaccharides stimulate leukocytes to produce cytokines in whole-cell cultures of human peripheral blood and in mouse splenocytes [13,14]. Through decreasing pro-inflammatory cytokine levels dependent on extracellular signal-regulated kinase, *Sparassis crispa* polysaccharides exhibited antiallergic effect in both *in vivo* and *in vitro* models [15]. Encouragingly, it has been successfully confirmed that *Sparassis crispa* extracts show positive activity on stroke through the activation of the AKT/endothelial nitric oxide synthase (eNOS) pathway in the brain of stroke-prone, spontaneously hypertensive (SHRSP) mice, which suggests its neuro-protective potential [16].

In the present study, polysaccharides obtained from *Sparassis crispa* were purified and characterized. Its neuro-protective effect and its underlying mechanisms were investigated on l-Glutamic acid (l-Glu)-induced damages on differentiated rat pheochromocytoma (DPC12) cells. Our data revealed that the purified polysaccharides improved cell viability and mitochondrial function, and restored the abnormal expression of apoptosis-related proteins. All these findings demonstrated that mitochondria-related pathways are essential for the neuro-protective effects of *Sparassis crispa* polysaccarides against l-Glu-induced toxicity in DPC12 cells.

## 2. Results

### 2.1. Purification and Characterization of Sparassis crispa Polysaccharides

**Figure 1:**
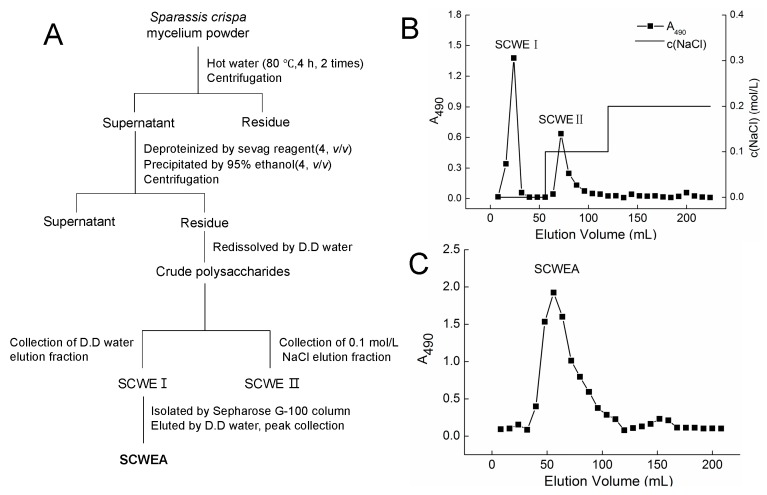
The purification of polysaccharides isolated from *Sparassis crispa*. (**A**) Scheme for extraction and isolation of polysaccharides from *Sparassis crispa*; (**B**) DEAE-52 cellulose anion exchanged the chromatogram of the crude polysaccharides. SCWE I and SCWE II were eluted by double-distilled (D.D.) water and 0.1 mol/L NaCl at a flow rate of 1.0 mL/min, respectively; (**C**) The crude polysaccharide SCWE I was further purified via Sepharose G-100, and SCWEA was obtained.

Polysaccharides obtained from the *Sparassis crispa* were separated by anion exchange chromatography (DEAE-cellulose column) (Figure 1A). Two fractions were eluted by double-distilled (D.D.) water and 0.1 M NaCl (Figure 1B), and they were named SCWE I and SCWE II, respectively. SCWE I was further purified by a gel permeation chromatography system Sepharose G-100, and the purified polysaccharides were named SCWEA (Figure 1C), which were applied in further experiments.

According to the high performance liquid chromatography (HPLC) data, rhamnose (Rha), mannose (Man) and galactose (Gal) were contained in SCWEA with the molar ratio of 10:2:1, and the molecular weight of SCWEA was 75 kDa (Table 1). The particle diameter of SCWEA was 88.9 nm (Table 1).

**Figure 2:**
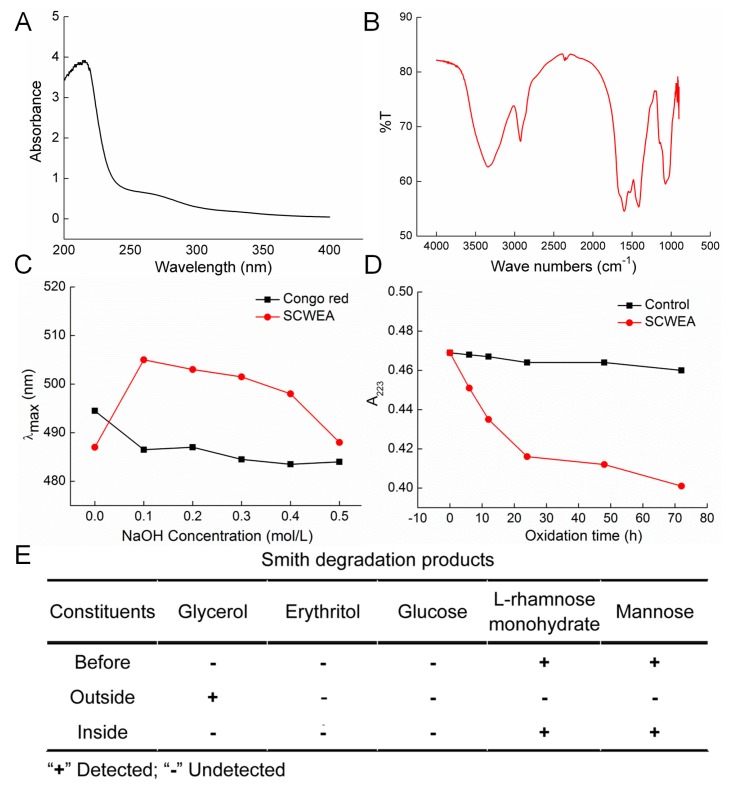
(**A**) Ultraviolet spectra of SCWEA; (**B**) Fourier transform infrared spectroscopy spectrum of SCWEA; (**C**) Congo red test on SCWEA; (**D**) Periodate oxidation time course; (**E**) Products of Smith degradation of SCWEA were detected using HPLC.

According to the UV spectra, no absorbance peak was caught at the wavelengths of 260 nm and 280 nm, which suggested no contamination of nuclear acid and proteins (Figure 2A). In the fourier transform infrared spectroscopy (FTIR) spectra, a strong hydroxyl absorption peak (3400 cm^−1^), a weak C–H absorption peak (2930 cm^−1^), and a C=O characteristic absorption peak (1650 cm^−1^) were observed (Figure 2B). Absorption between 950~1200 cm^−1^ suggested the existence of C–O–C and C–O–H. The red shift of SCWEA in λ_max_ compared with congo red solution suggested a triple helix conformation of SCWEA (Figure 2C). Periodate oxidation–Smith degradation was performed to detect the linkage model of glucose within SCWEA (Figure 2D,E). The HPLC method was performed to analyze the hydrolysates after Smith degradation. After hydrolysis, rhamnose and mannose were observed inside the sack fraction while glycerol other than erythritol exists outside (Figure 2E). In addition, 102 µmol periodate was consumed with 27.5 µmol of formic acid generated during NaIO_4_ oxidation on SCWEA.

**Table 1:** Molecular weight and particle size.

**Molecular weight ^1^**	**Mr (kDa)**	**molar ratio, mol %**
	75	96.7
**Particle size ^2^**	**Z-Average (r.nm) ^3^**	**PdI ^4^**
	44.45	0.306

^1^ Molecular weight determined by HPLC; ^2^ Particle size determined by DLS; ^3^ r: radius of SCWEA; ^4^ PdI: polydispersity.

### 2.2. The Effect of SCWEA against l-Glu Induced Cell Damage in DPC12 Cells

**Figure 3:**
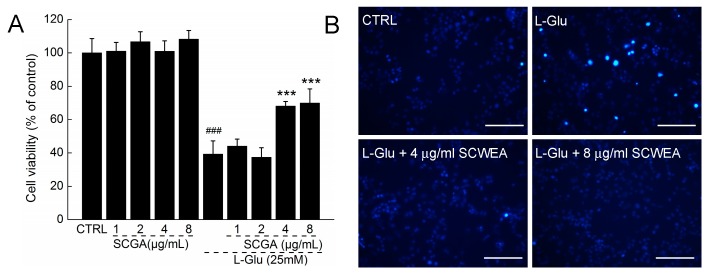
The neuro-protective effect of SCWEA against l-Glu-induced cell damage in DPC12 cells. Cells were pretreated with SCWEA for 3 h, and then co-incubated with or without 25 mM l-Glu for 24 h. Compared with l-Glu-treated cells, SCWEA enhanced cell viability (**A**) and reduced nuclear apoptosis rate (20×; Bar: 100 µm) (**B**). Data were expressed as a percentage of corresponding control cells and means ± S.D. (*n* = 6). ### *p* < 0.001 *versus* control cells (CTRL); *** *p* < 0.001 *versus* l-Glu-exposed cells.

After incubation with various concentrations of SCWEA and/or 25 mM l-Glu for 24 h, the viability of DPC12 cells was determined by 3-(4,5-cimethylthiazol-2-yl)-2,5-diphenyl tetrazolium bromide (MTT) assay. Results showed that there was an approximately 60.8% reduction of cell viability (*p* < 0.001; Figure 3A) in l-Glu-treated DPC12 cells, whereas SCWEA alone showed minimal affect on cell viability. Pre-treatment with SCWEA at doses of 4 and 8 µg/mL improved nearly 28.9% (*p* < 0.001) and 30.8% (*p* < 0.001) of the viability of l-Glu-explored cells (Figure 3A). SCWEA at doses of 4 and 8 mg/mL strongly reversed the apoptosis rate in 25 mM l-Glu-explored DPC12 cells, as indicated by the decreasing blue fluorescence intensity of nuclear staining with Hoechst 33342 (Figure 3B).

### 2.3. The Effects of SCWEA on Intracellular Calcium Concentration and Reactive Oxygen Species (ROS) Levels

**Figure 4:**
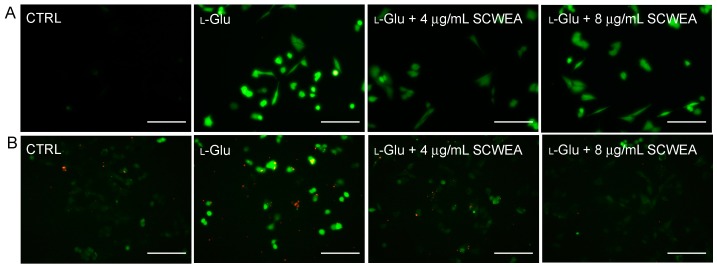
SCWEA suppressed Ca^2+^ influx (**A**) and intracellular reactive oxygen species (ROS) accumulation (**B**) in l-Glu-exposed DPC12 cells (20×; Bar: 100 µm). Cells were pretreated with 4 and 8 µg/mL SCWEA for 3 h, followed by exposure to 25 mM l-Glu for another 12 h. The intracellular levels of Ca^2+^ and ROS were detected by Fluo-4-AM and DCFH-DA staining, respectively. The experiments were repeated three times.

Results obtained from 2,7-dichlorofluorescin diacetate (DCFH-DA) staining revealed that 12 h l-Glu incubation led to an accumulation of intracellular ROS, as suggested by the increase of DCF fluorescence. Lower green fluorescence noted in SCWEA–pre-treated DPC12 cells indicated that SCWEA effectively reduced intracellular ROS levels after 12 h of co-incubation (Figure 4A).

Fluo-4-AM staining was applied to examine the intracellular calcium concentration in DPC12 cells. An extremely high green fluorescence was observed in 25 mM l-Glu-explored cells, which was significantly relieved by 3 h of pre-treatment of SCWEA (4 and 8 µg/mL) (Figure 4B).

### 2.4. The Effects of SCWEA on Mitochondrial Function

**Figure 5:**
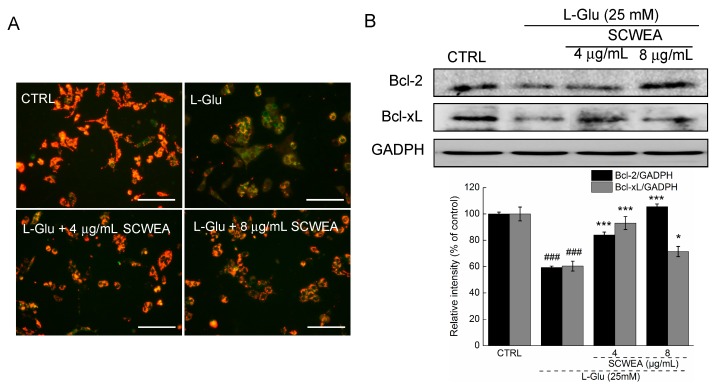
The positive regulatory effects of SCWEA on mitochondrial function. DPC12 cells were pretreated with 4 and 8 µg/mL SCWEA for 3 h, followed by exposure to 25 mM l-Glu for 12 or 24 h. (**A**) SCWEA restored l-Glu-induced MMP loss. The changes of MMP were determined by JC-1 staining (20×; Bar: 100 µm). Red fluorescence indicates healthy cells with high MMP, whereas green fluorescence indicates apoptotic or unhealthy cells with low MMP. The experiments were repeated three times; (**B**) SCWEA enhanced the expressions of B-cell lymphoma 2 (Bcl-2) and B-cell lymphoma-extra large (Bcl-xL) in l-Glu-exposed DPC12 cells. Quantification data were normalized by GAPDH. Data were expressed as a percentage of corresponding control cells and means ± S.D. (*n* = 3). ### *p* < 0.001 *versus* control cells; * *p* < 0.05 and *** *p* < 0.001 *versus* l-Glu-exposed cells.

Mitochondrial membrane potential was known to play an important role in regulating the intrinsic and extrinsic apoptosis pathways in cells. Changes of MMP in DPC12 cells were monitored by a JC-1 (5,5′,6,6′-tetrachloro-1,1′,3,3′-tetraethylbenzimidazolylcarbocyanine iodide) molecular probe, and the fluorescent shift from red to light green reflected the depolarization of MMP [17]. SCWEA at doses of 4 and 8 µg/mL strongly restored MMP in l-Glu-treated cells (Figure 5A). In addition, a striking reduction in the expressions of Bcl-2 and B-cell lymphoma-extra large (Bcl-xL) was noted in l-Glu-treated cells. The 8 µg/mL of SCGA pre-treatment resulted in 24.7% and 46.1% enhancement of Bcl-2 and Bcl-xL expression compared with that of l-Glu-treated cells (Figure 5B).

### 2.5. Protein Kinase B (AKT) Contributing to SCWEA-Mediated Neuro-Protective Effect

**Figure 6:**
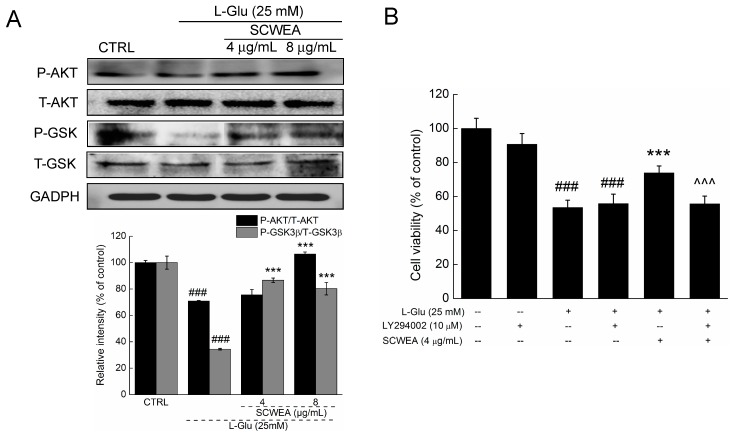
AKT/GSK3β pathway contributes SCWEA-mediated neuro-protective effect. DPC12 cells were pre-treated with 4 and 8 µg/mL SCWEA for 3 h, followed by exposure to 25 mM glutamate for 24 h. (**A**) The activation of AKT and GSK-3β was detected via Western blot. Quantification data were normalized by corresponding T-AKT and T-GSK3β, respectively; (**B**) Protective effects of SCWEA against l-Glu on cell viability were abolished by LY294002 pre-treatment. DPC12 cells were pre-treated with 10 µM LY294002 for 30 min, followed by treatment with 4 µg/mL SCWEA for 3 h and exposure to 25 mM l-Glu for 24 h. Data were expressed as a percentage of corresponding control cells and means ± S.D. (*n* = 6). ### *p* < 0.001 *versus* control cells; *** *p* < 0.001 *versus* l-Glu-exposed cells; ^^^ *p* < 0.001 *versus* SCWEA and l-Glu co-treated cells.

Reduced phosphorylation of AKT (P-AKT) and glycogen synthase kinase 3 β (P-GSK-3β) was observed in l-Glu-exposed DPC12 cells after 24 h of treatment (*p* < 0.001; Figure 6A). SCWEA 3 h pre-treatment replenished the activation of AKT and GSK-3 β in l-Glu-challenged DPC12 cells (*p* < 0.001; Figure 6A). No significant changes in the expressions of total AKT (T-AKT) and total GSK-3β (T-GSK-3β) were observed (Figure 6A).

Furthermore, the neuro-protective effect of SCWEA was significantly abrogated by 30 min of pre-treatment with 10 µM LY 294002, an AKT inhibitor (73.8% ± 4.1% *versus* 55.7% ± 4.6%; *p* < 0.001; Figure 6B). Taken together, these findings suggest the activation of AKT mediates neuro-protection of SCWEA in DPC12 cells.

## 3. Discussion

Our study demonstrated, for the first time, the neuro-protective effect of purified polysaccharides separated from *Sparassis crispa* (SCWEA). The characteristic structure of SCWEA was analyzed by FTIR, congo red and periodate oxidation-Smith degradation. Generation of 27.5 µmol formic acid during NaIO_4_ oxidation suggested the existence of (1→6) linkages; meanwhile, the low yield of formic acid suggested that these (1→6) linkages do not belong to the main chain. Minimal consumption of periodate (0.85 mol per glycosyl) indicated the existence of (1→3) linkages, which shall not consume any periodate during oxidation. The remaining rhamnose and mannose in the hydrolysates after Smith degradation further confirmed the existence of (1→3) linkages. No (1→4) linkages existed within SCWEA, as indicated by the absence of erythritol in hydrolysates. SCWEA may structuralize with (1→2) linkages since over two more folds of periodate consumption than formic acid generation was observed. Collectively, SCWEA contains (1→3)-linked Rha in the backbone, as well as (1→2) linkages and (1→6) linkages in the side bone. Combined with the results obtained from congo red test, the conformation of SCWEA is a triple helix rather than a random coil.

**Figure A1:**
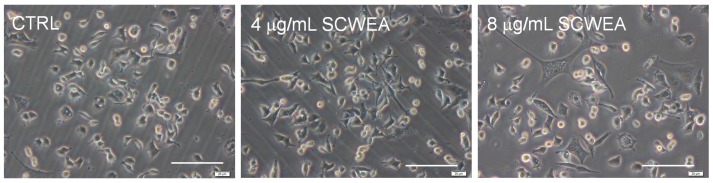
The regulatory effects of SCWEA on the morphology of PC12 cells. PC12 cells were exposed to 4 and 8 µg/mL SCWEA for 24 h and the morphologies were detected via microphotograph. 10×, scale bar: 50 µm.

The present data has successfully confirmed the neuro-protective effect of SCWEA against l-Glu-induced cell damage via improving cell viability and reducing nuclear apoptotic rate. Preliminarily, data also displayed that SCWEA alone successfully induced neurogenesis and differentiation in PC12 cells (Figure A1). The changed parameters caused by central nervous system stress, such as the shutdown of nerves, result in high levels of glutamate, which may in turn lead to cell apoptosis [18]. Mitochondrial dysfunction is involved in l-Glu-caused neurotoxicity. As the major sources of cellular ATP and a hub for Ca^2+^ signaling, mitochondria are considered as a center of intracellular energy metabolism [19]. Mitochondrial Ca^2+^ is a positive effector of ATP synthesis, yet Ca^2+^ overload leads to the production of free radicals and the opening of mPTP, which, taken together, result in mitochondrial depolarization, matrix solute loss and cytochrome c release [20]. On the other hand, the overproduction of ROS is also linked to the opening of mPTP [21]. SCWEA pre-treatment significantly prevented intracellular Ca^2+^ and ROS accumulation, and mitigated MMP dissipation. Our data further revealed that SCWEA enhanced l-Glu-suppressed expressions of Bcl-2 and Bcl-xL, the anti-apoptotic members of the Bcl-2 family that maintain normal mitochondrial function and prevent mitochondrial outer membrane permeabilization. Bcl-2 is an integral membrane protein, and the expression of Bcl-2 is known as a hallmark of mitochondria dysfunction–associated cell death [22]. Altogether, the mitochondrial apoptotic pathway may be mainly involved in SCWEA-mediated neuro-protection against L-Glu-caused DPC12 cell damage.

As reported, liquiritin modulated AKT and its downstream element GSK-3β to relieve l-Glu-induced cell damage in DPC12 cells. The AKT signaling molecule is involved in cell differentiation, proliferation, survival and apoptosis [23]. The activation of AKT can suppress several pro-apoptotic proteins such as Bcl-2 family members and some signal molecules such as GSK-3β [24]. Micro-vesicles released by mouse bone marrow-derived mesenchymal stem cells were reported to protect PC12 cells from l-Glu-induced excitotoxicity via upregulating AKT phosphorylation and Bcl-2 expression [25]. GSK-3β participates in apoptosis in several cell types, and it is known as an upstream regulator of programmed cell death. GSK-3β promotes activation and translocation of Bax, which is related to mitochondrial dysfunction [26]. In addition, GSK-3β is considered a critical factor in mediating cytosolic leakage of cytochrome c from mitochondria, and phosphorylated GSK-3β inhibits the opening of mPTP [27]. Furthermore, 25-hydroxycholesterol could induce mitochondria-dependent apoptosis via the activation of GSK-3β in PC12 cells [28]. l-Glu-induced suppression of AKT and subsequent dephosphorylation of GSK-3β, as we demonstrated, would be expected to provide a mitochondria-related pro-apoptotic stimulus. Combining literature study with our present results, we proposed that the neuro-protective effect of SCWEA in l-Glu-treated neurons is mainly dependent on the restoration of AKT and the subsequent shutdown of the mitochondrial apoptotic pathway.

**Figure A2:**
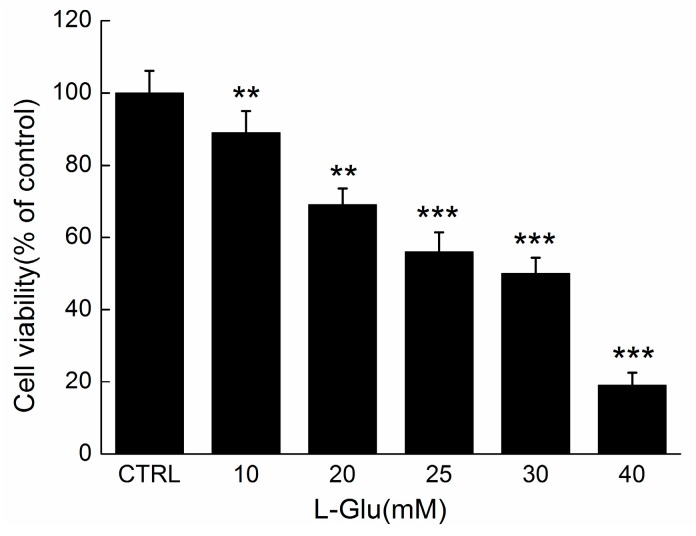
Effects of various concentrations of l-Glu on the viability of DPC12 cells. DPC12 cells were exposed to 10–40 mM of l-Glu for 24 h. Data were expressed as a percentage of corresponding control cells and means ± S.D. (*n* = 6). ** *p* < 0.01, *** *p* < 0.001 *versus* control cells.

Concerns were particularly taken with the concentration of glutamate in the present study. Although the highest concentration of glutamate in the brain is 10–12 mM, the potential glutamate level for excitotoxicity in normal cerebrospinal fluid (CSF) is at the remarkably low level of 100–200 nM [29]. For *in vitro* experiments, 20 to 25 mM glutamate were used to induce damage of DPC12 cells [30,31]. Our preliminary experiments tested the cytotoxic effects of l-Glu ranging from 10 to 50 mM, and eventually 25 mM glutamate was utilized to establish the neuro-toxic DPC12 cell model (Figure A2).

In summary, SCWEA purified from *Sparassis crispa* exhibits neuro-protective activity against l-Glu-induced DPC12 cell damage, which is, at least partially, related to the activation of AKT and the mitochondrial pathway. Our study suggests that SCWEA could be a potential therapeutic or modulating agent for neurodegenerative damage.

## 4. Materials and Methods

### 4.1. Submerge Fermentation of Sparassis crispa and Crude Extract Preparation

*Sparassis crispa* (SC031; CCTCC No.M2015275) was cultured in a hundred-liter full-automatic fermentor (BaoXing Bioscience Company, Shanghai, China) by using a defined liquid medium which contains: 20 g/L sucrose, 5 g/L yeast extract powder, 1 g/L MgSO_4_·7H_2_O and 0.25 g/L vitamin B1. *Sparassis crispa* mycelium obtained from submerged fermentation was extracted at 80 °C for 4 h in D.D. water and the process was repeated two times. After being centrifuged, the supernatant (SCWE) was sequentially concentrated and freeze-dried for further experiments.

### 4.2. Polysaccharide Purification and Characterization

#### 4.2.1. Purification of *Sparassis crispa* Polysaccharides

Sevag reagent (*v*(*n*-butanol):*v*(chloroform) = 1:4, 50 mL) was used to remove proteins in SCWE. Four-fold ethanol was added to the supernatant and placed at 4 °C overnight [32]. Furthermore, the dissolved precipitation in D.D. water was subjected to DEAE-52 cellulose anion exchange column (2.6 cm × 35 cm; Whatman, Bucks, UK). The column was eluted with D.D. water followed with 0.1 and 0.2 mol/L NaCl at a flow rate of 1 mL/min. The polysaccharide fraction was collected and detected using phenol-sulfuric acid method. Briefly, samples (1 mL) mixed with 1 mL phenol solution (5%) were added into 20 mL tube. Ten minutes after 5 mL sulfuric acid addition, the tubes were cooled down at 30 °C for 20 min. The absorbance was spectrophotometrically measured at a wavelength of 490 nm [33]. Further purification was performed via a gel permeation chromatography system Sepharose G-100 (Pharmacia, Uppsala, Sweden). The column was eluted with D.D. water at a flow rate of 0.5 mL/min. The fractions (10 mL each) were collected, detected and then freeze-dried. The purified polysaccharides were named SCWEA (Figure 1A).

#### 4.2.2. Molecular Weight and Particle Size Measurements

The molecular weight was analyzed by LC-10ATvp HPLC system (Shimadzu, Tokyo, Japan), which is equipped with a TSK-GEL G4000PWXL column (Tosoh Co., Tokyo, Japan) and an Alltech 2000ESELSD (Shimadzu, Tokyo, Japan). D.D. water was served as the mobile phase, the flow rate was 0.45 mL/min, aerosol level was 60%, drift tube temperature was 120 °C and nebulizing nitrogen pressure was 25 psi. The dextran standards were used to create a calibration curve [34]. The particle size of polysaccharides in deionized water was detected by Dynamic light scattering (DLS) using a Malvern Zetasizer Nano (ZS90, Malvern Instruments Ltd., Malvern, UK). The sample was dissolved in the D.D. water at a concentration of 1 mg/mL, and there were 10 runs performed in each procedure.

#### 4.2.3. Ultraviolet (UV) Spectra Measurement

UV-2401PC UV-Vis recording spectrophotometer (Shimadzu Corporation, Tokyo, Japan) was applied to obtain the spectra of purified polysaccharide. Each spectrum was at the average of five parallel scanning from 200 to 400 nm. The scan interval was 0.2 nm and the width of entrance slit was 12 nm.

#### 4.2.4. Fourier Transform Infrared Spectroscopy (FTIR) Determination

A 4 mg sample was ground thoroughly with 150 mg KBr, and its average transmission spectra (*n* = 100) were recorded via a IRPrestige-21 FTIR spectrometer (Shimadzu, Tokyo, Japan) at the wavelength ranging from 900 to 4000 cm^−1^.

#### 4.2.5. Congo Red Test

Congo red test was performed to detect the chain conformation of polysaccharides in aqueous solution, in which a red shift in the light absorption maximum λ_max_ was attributed to the triple helices of polysaccharide chains [35]. Sample aqueous solution at 1 mg/mL containing 81 µM Congo red was treated with 1 M NaOH at various concentrations ranging from 0 to 0.5 M. Visible light absorption spectrum was obtained over the range from 200 to 900 nm at room temperature with a UV-2401PC UV-Vis spectrophotometer and deionized water which were considered as the blank control.

#### 4.2.6. Monosaccharides Analysis

The polysaccharides (20 mg) were hydrolyzed with 1 M H_2_SO_4_ (1 mL) at 105 °C for 6 h in a sealed glass tube, and then the pH was adjusted to be neutral with BaCO_3_. The solution was then centrifuged at 4000× *g* for 10 min to separate the hydrolysates, which were further analyzed by using the HPLC/ELSD (Evaporative Light-Scattering Detector) system. The chromatograph was fitted with a Prevail™ ES carbohydrate analysis column (Alltech Associates, Inc., Deerfield, IL, USA), which was eluted with 75% acetonitrile (Sigma-Aldrich, St. Louis, MO, USA) at a flow rate of 1.0 mL/min. The results were compared with the monosaccharide standards: d-glucose, l-rhamnose (Rha), d-xylose, d-galactose (Gal), d-mannose (Man) and l-arabinose (Sigma-Aldrich, USA) [34].

#### 4.2.7. Periodate Oxidation-Smith Degradation Reaction of Polysaccharides

Similar to the previous study [36], 20 mg polysaccharide was dissolved in 15 mM NaIO_4_ (25 mL, pH 4) at 4 °C in darkness. NaIO_4_ in different concentrations were used to create a calibration curve to calculate the HIO_4_ consumption, while the formic acid production was determined by titration. After 48 h dialyzing against D.D. water, the dialysate was concentrated and incubated with potassium borohydride (70 mg) overnight at room temperature. After adjusting pH value to 7.0 by acetic acid, the solution was dialyzed against D.D. water for another 24 h. The 3 mL sample was detected by HPLC/ELSD system. The rest of product was hydrolyzed with 1 M H_2_SO_4_ at 25 °C for 40 h, and the pH value was adjusted to 7.0 by BaCO_3_. The solution was centrifuged at 4000× *g* for 10 min to separate the hydrolysates which were further analyzed by HPLC/ELSD system.

### 4.3. Cell Culture

PC12 cells (obtained from ATCC, Manassas, VA, USA; CRL-1721; passages < 10) grew in Dulbecco’s modified Eagle medium (DMEM; Invitrogen, Grand Island, NY, USA) which was supplemented with 5% horse serum (HS; Invitrogen, USA), 10% fetal bovine serum (FBS; Invitrogen, USA), penicillin (100 units/mL), and streptomycin (100 µg/mL) (Invitrogen, USA), under a humidified atmosphere containing 5% CO_2_ at 37 °C. The culture medium was changed every three days. PC12 cells were differentiated for 48 h with 50 ng/mL nerve growth factor (NGF; Sigma-Aldrich, USA) which was dissolved in DMEM medium containing 1% FBS, 1% HS and 100 U/mL of penicillin/streptomycin.

### 4.4. Cell Viability Analysis

A quantitative colorimetric assay with MTT (Sigma-Aldrich, USA) was applied to measure cell viability. DPC12 cells were seeded in 96-well plates at 2 × 10^4^ cells/well. Cells were treated with SCWEA (1–8 µg/mL) alone for 24 h, or pretreated with SCWEA (1–8 µg/mL) for 3 h and exposed to 25 mM l-Glu for another 24 h. In another separated experiment, cells were pretreated with 10 µM LY294002 for 30 min, and exposed to 4 µg/mL SCWEA for 3 h, followed with another 24 h co-incubation with 25 mM l-Glu. After incubation with MTT solution (0.5 mg/mL) for 4 h at 37 °C in darkness, 100 µL dimethyl sulfoxide (DMSO) was added to dissolve crystals. A micro-plate reader (Bio-Rad, Hercules, CA, USA) was used to detect the absorbance at 540 nm. Viability values of treated cells were expressed as a percentage of that of corresponding control cells.

### 4.5. Cellular Morphology Analysis

Nucleus morphological alterations were analyzed by Hoechst 33342. PC12 cells (1 × 10^5^) were seeded into each well in a six-well plate. After differentiation, cells were pre-treated with 4 and 8 µg/mL SCWEA for 3 h, followed with 12 h co-incubation with 25 mM l-Glu. Then cells were incubated with Hoechst 33342 (5 µg/mL; Sigma-Aldrich, USA) for 15 min at 37 °C in darkness. After being washed with PBS, the fluorescence intensity in the nucleus was photographed by a fluorescent microscope (20×; CCD camera, TE2000, Nikon, Tokyo, Japan).

### 4.6. Measurement of ROS

The production of intracellular ROS was measured via DCFH-DA (Nanjing Jiancheng, Nanjing, China). The DCFH-DA passively entered the cell where it reacted with ROS to form the highly fluorescent compound dichlorofluorescein (DCF). Briefly, DPC12 cells were exposed to 4 and 8 µg/mL SCGA, and co-incubated with l-Glu (25 mM) for 12 h. Treated cells were incubated with 10 µM DCFH-DA at 37 °C for 30 min. The fluorescent color was photographed by a fluorescent microscope (20×; CCD camera) after being washed with PBS three times.

### 4.7. Measurement of Intracellular Calcium Concentration ([Ca^2+^]i)

DPC12 cells were seeded in a six-well plate (1 × 10^5^ cells/well). On the next day, cells were treated with 4 and 8 µg/mL SCGA for 3 h prior to exposure to 25 mM l-Glu for 12 h. Then, the supernatant was removed, and cells were incubated with 5 µM Fluo-4-AM (Invitrogen, USA) for 30 min at 37 °C in darkness. After three washes, moderate PBS was added to cover the cells, and then cells were observed through fluorescence microscope (20×; CCD camera).

### 4.8. Mitochondrial Membrane Potential (MMP) Analysis

DPC12 cells (1 × 10^5^) were seeded into each well in a six-well plate. Overnight cells were pre-treated with 4 and 8 µg/mL SCWEA for 3 h, followed with 12 h co-incubation with 25 mM l-Glu. Treated cells were incubated with 2 µM JC-1 (Sigma-Aldrich, USA) at 37 °C for 10 min. Fluorescent microscope (20×; CCD camera) was applied to record the fluorescent color in each group.

### 4.9. Western Blot

DPC12 cells were pre-treated with 4 and 8 µg/mL SCWEA for 3 h, and followed with 24 h co-incubation with 25 mM l-Glu. Cells were lysed by radioimmunoprecipitation assay (RIPA) buffer (Sigma-Aldrich, USA) which contains 2% phenylmethanesulfonyl fluoride (PMSF; Sigma-Aldrich, USA) and 1% protease inhibitor cocktail (Sigma-Aldrich, USA). Proteins were separated via 12% SDS-PAGE gel and transferred electrophoretically onto nitrocellulose membranes (Bio Basic, Amherst, NY, USA). The transferred membranes were then blotted with antibodies as follows: Bcl-2, Bcl-xL, T-AKT, P-AKT, T-GSK-3β, P-GSK-3β and glyceraldehyde-3-phosphate dehydrogenase (GAPDH) (1:1000; Abcam, Cambridge, UK) at 4 °C overnight, followed by incubation with horseradish peroxidase-conjugated secondary antibodies (Santa Cruz, CA, USA). Chemiluminescence was detected by using ECL detection kits (GE Healthcare, Bucks, UK). The intensity of the bands was quantified by scanning densitometry using software Image J (National Institutes of Health, Bethesda, MD, USA).

### 4.10. Statistical Analysis

All data were presented as mean ± standard deviation (S.D.). Data were evaluated by one-way analysis of variance (ANOVA) to detect statistical significance, followed by *post-hoc* multiple comparisons (Dunn’s test) by using SPSS 16.0 software (SPSS Inc., Chicago, IL, USA). Values of *p* < 0.05 were considered to be significant.
